# The bilateral limb deficit (BLD) phenomenon during leg press: a preliminary investigation into central and peripheral factors

**DOI:** 10.1186/s13102-021-00321-0

**Published:** 2021-08-13

**Authors:** Emily Whitcomb, Oscar Ortiz, Jacqueline Toner, Usha Kuruganti

**Affiliations:** Andrew and Marjorie McCain Human Performance Laboratory, Faculty of Kinesiology, University of New Brunswick, Fredericton, NB E3B 5A3 Canada

**Keywords:** Bilateral limb deficit phenomenon, Surface electromyography, Electroencephalography, Leg press, Movement related cortical potential

## Abstract

**Background:**

The bilateral limb deficit (BLD) phenomenon suggests that lower forces are produced with bilateral limb contractions compared to the summed force produced when the same muscles are contracted unilaterally. While interhemispheric inhibition has been suggested as a cause of BLD, the origin of the deficit is yet to be determined. The aim of this study was to investigate central and peripheral factors responsible for the BLD during leg press using surface electromyography (EMG) and electroencephalography (EEG).

**Methods:**

Fourteen adults (age = 23.7 ± 4.7 years old) completed bilateral (BL), unilateral left (UL) and unilateral right (UR) isometric leg press exercises. Bilateral limb ratio (BLR) was calculated similar to previous studies and surface EMG from three muscles of the quadriceps femoris (vastus lateralis, vastus medialis and rectus femoris) was used to measure the level of muscle activation. Movement related cortical potentials (MRCPs) over the left and right motor cortex areas (C3 and C4, respectively) were used to assess brain activity asymmetries reflecting central factors.

**Results:**

No significant difference was noted in the mean BLR (BLR = 94.8%), but a subset of ten participants did demonstrate a BLD (BLR = 81.4%, *p* < 0.01). Mean differences in relative activation were found among the three quadricep muscles (*p* < 0.001) with the right VM having significantly higher amplitude for the unilateral right (0.347 ± 0.318 mV) and bilateral right (0.436 ± 0.470 mV) conditions, respectively) than either the VL or RF (*p* < 0.05). The VL had significantly lower amplitudes in all conditions (0.127 ± 0.138 mV; 0.111 ± 0.104 mV; 0.120 ± 0.105 mV; 0.162 ± 0.147 mV for unilateral left, bilateral left, unilateral right, and bilateral right, respectively). However no overall significant differences were noted between bilateral and unilateral conditions. No significant differences in MRCPs were observed between brain activity of the C3 and C4 electrodes in any of the conditions.

**Conclusion:**

While the sample size was low, this exploratory study noted the presence of BLD however the results did not provide evidence of significant limitations in either the EMG or EEG data.

## Background

Evidence suggests that lower forces are produced with bilateral limb contraction when compared to the summed force produced when the same homologous muscles are contracted unilaterally [21, 32]. This phenomenon, termed as bilateral limb deficit (BLD), has been exhibited in both upper and lower limbs, however the magnitude of the deficit is typically larger in lower limbs [22]. BLD occurs similarly in males and females [16, 18, 19], but it has been shown to be sensitive to limb dominance and training interventions [2, 8, 18, 39] It has been demonstrated that specificity training can reduce BLD, for example, training under unilateral and bilateral conditions can increase unilateral and bilateral strength, respectively [18, 31, 39].

Despite the established force deficit, the source of BLD is still poorly understood. Exploring the underlying roots of the deficit is important to understand neuromuscular function and how and why it reflects in tasks which use both limbs simultaneously. Investigating the source of this inhibition will help to understand the effect of the deficit and its functional implications including muscle imbalance and coordination.

Two primary theories for the cause of the BLD have been suggested, the postural stability theory and the neural inhibition theory [21]. The postural stability theory postulates that postural stability requirements of the exercise studied may be the cause of the deficit [7, 22]. This was further supported by evidence demonstrating that multi-jointed lower body exercises, particularly those involving large muscles and high force generation, require more postural stability and exhibit a greater deficit [20].

Magnus and Farthing [22] examined the impact of postural stability on the BLD and investigated the effects of the BLD during leg press and handgrip exercise. They found BLD present only during leg press, however they noted that the muscle activation was not significantly different between unilateral and bilateral conditions for either exercise. They did note, however that core muscle activation was greater during leg press compared to handgrip providing some support to their hypothesis that those exercises requiring more postural stability (in their case the leg press) would have a greater BLD than an exercise requiring less postural stability (e.g. handgrip).

The neural inhibition theory, conversely, has received more attention. This theory proposes that unilateral contractions are caused by complex interactions between specific areas of the cortex and that the bilateral deficit is a result of neural inhibition of motor drive by neural activity in the contralateral motor tract [36]. Surface electromyography (EMG) obtained through RMS calculations measures the extent of the neural commands sent to the muscle. Although some work has had mixed results regarding the relationship between force and muscle activity [3, 9, 8, 17, 19], there is evidence suggesting neural mechanisms are behind BLD. Early research [27] suggested that the deficit could be related to inhibitory spinal reflexes, which occur when the neural control for one limb is affected when the opposite limb is simultaneously activated. It is possible that afferent sensory information from one limb may inhibit the control of the motor neurons acting on the contralateral limb [13]. Furthermore, a study looking at BLD in plantar flexor muscles also suggested reduced motor neuron excitability during bilateral contraction may also contribute to BLD [12].

Despite this evidence, making conclusions about where these changes happen in the neural circuitry is limited, as EMG only provides a final snapshot of the overall neural commands sent to the muscle. Voluntary movement of the limbs is attained through the corticospinal tract, made of upper motor neurons (UMN), originating mostly from the primary motor cortex, which in turn synapse with interneurons and lower motor neurons (LMN) that lead to the neuromuscular junctions responsible for the contraction and relaxation of skeletal muscle. To disentangle whether this neurological change causing BLD occurs in the central UMN or in the peripheral interneurons and LMN, experiments have used measures of brain activity such as electroencephalography (EEG) to investigate the potential central neurological origin for BLD.

EEG can be an effective method of measuring brain activity during human movement [37]. During maximal voluntary contractions, researchers have described the preparatory brain activity that occurs prior to the onset of movement is demonstrated through movement-related cortical potentials (MRCPs) that can be measured through EEG. MRCPs are composed of two distinct components. The first component is termed the Bereitschaftspotential (or Readiness Potential, RP), which is classified as a slow negative shift that occurs between onset of movement and is related to the MRCP peak amplitude [34, 37]. The second component is called the Negative Slope (NS’) which occurs between 500 ms before the onset of movement and movement onset. The Motor Potential (MP) falls within the NS’ and occurs at the onset of movement and corresponds to the peak amplitude of the MRCP. MRCPs have been investigated in the primary motor cortex area (electrodes C3 and C4 for the left and right cortex, respectively) during unilateral and bilateral handgrip contractions [24, 25]. Their results indicated that a bilateral deficit in both force production and EMG was associated with a reduction in MRCPs, indicating that the bilateral handgrip contraction produced less force and EMG activity than the unilateral handgrip contraction because of a mechanism of interhemispheric inhibition. Interhemispheric inhibition is thought to occur when the activity in one hemisphere of the brain affects the activity in the opposite hemisphere while both are concurrently activated, thereby decreasing neural drive to the muscles [24, 40, 41]. However, there remain few studies that have examined brain activity simultaneously with EMG and force to study BLD, and this effect has not been shown during lower limb movements.

The purpose of the current study was to investigate the underlying cause of the BLD phenomenon in active, young adults in the lower limbs. Force output was recorded in parallel with surface EMG and EEG data during unilateral and bilateral leg press exercises using an isokinetic dynamometer. It was hypothesized that (1) the bilateral force output will be less than the sum of the unilateral force output during the leg press, (2) the unilateral muscle activity will support the discrepancies in force output, and (3) there will be differences in neuronal activity between the bilateral and unilateral leg press suggesting that the bilateral deficit is caused, at least in part, by the central nervous system.

## Methods

Fourteen healthy men (n = 5) and women (n = 9) participated in the present study. Participant characteristics are summarized in Table 1. A questionnaire was distributed prior to beginning the study and it was observed that all participants were right-leg dominant (determined by asking which leg they would kick a soccer ball with) and were considered active (i.e., they engaged in resistance training at least three times per week on a regular basis) but were not varsity athletes. All participants were provided a detailed overview of the study and written informed consent was obtained from each participant prior to testing. This study was approved by the University of New Brunswick Research Ethics Board and has been assigned the file number REB#2019-159.

**Table 1:** Participant characteristics

Characteristic	Male (n = 5)	Female (n = 9)	Total (n = 14)
Age (years)	27.2 ± 6.8	21.8 ± 1.3	23.7 ± 4.7
Height (m)	1.79 ± 0.06	1.66 ± 0.06	1.71 ± 0.09
Weight (Kg)	82.8 ± 9.3	69.7 ± 8.2	74.4 ± 10.5
Body mass index (BMI) (kg/m^2^)	25.7 ± 1.9	25.3 ± 3.1	25.5 ± 2.7
Thigh girth (cm)	61.5 ± 3.2	62.7 ± 6.3	62.3 ± 5.3
Right anterior thigh skinfold (mm)	12.0 ± 3.2	15.1 ± 4.0	14.0 ± 3.9
Right anterior patella skinfold (mm)	9.9 ± 3.9	14.6 ± 2.0	12.9 ± 3.6

### Instrumentation

Torque data for the unilateral and bilateral leg press was collected using an isokinetic dynamometer (Cybex Humac Norm, CSMI Inc., USA) with an attached closed kinetic chain adapter. The sampling frequency of the dynamometer was 100 Hz. A 32-channel wireless surface EMG system (Trentadue, OT Bioeletrronica, Italy) was used to record muscle activity during all maximal voluntary contractions (MVCs). The EMG system had a Common Mode Rejection Ratio (CMMR) of over 96 dB and a signal bandwidth of 10/500 Hz. The signals were sampled at a frequency of 2000 Hz, and an A/D converter resolution of 24 Bit, with a gain of 256. A dry, wireless EEG system (Cognionics Quick-30 Dry Electrode, Cognionics Inc., San Diego, CA, USA) was used to acquire brain activity during the leg press at a sampling frequency of 1000 Hz. To create a time-stamp for the MRCPs, a microcontroller (Arduino MEGA 2560, Arduino LCC, Italy) was used to send a trigger impulse to the EEG system when the participant reached 5 percent of their maximum torque production.

### Isometric strength testing

**Fig. 1:**
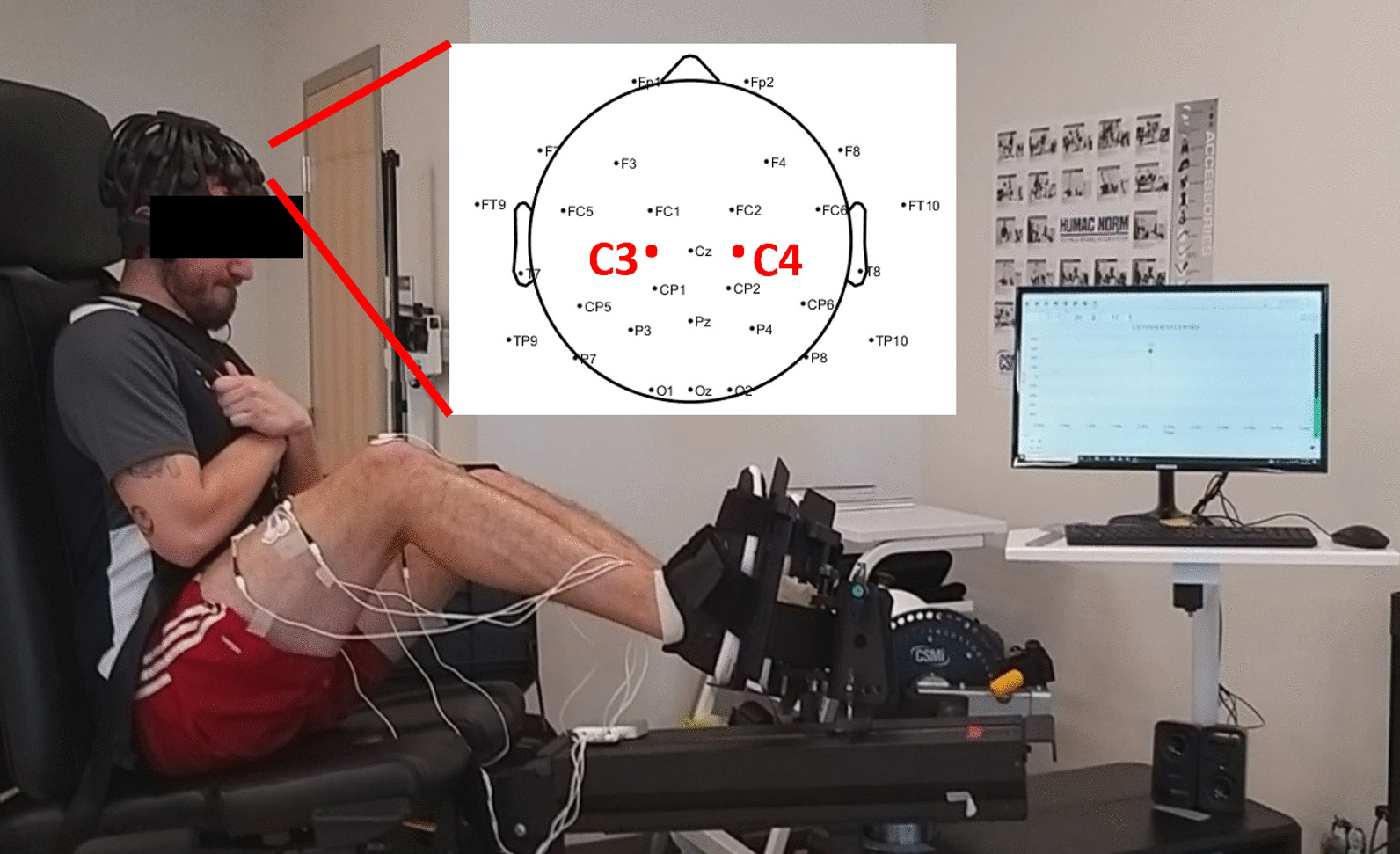
Experimental set up with EEG electrode markers indicated. Electrode placements on the left (C3) and right (C4) precentral cortex are indicated. Written consent was provided by participant

Participants were seated in an upright position on the Cybex (Fig. 1). The dynamometer was positioned at a self-selected back-angle (approximately 90°) and a horizontal translation (35–40°) to ensure comfort, and the closed kinetic chain adapter was set so that participant’s knees were at a 90° angle, measured using a goniometer. Hip angle varied as participant’s were able to adjust the back angle for comfort, but the angle was typically kept at approximately 90° (85–100°). Participants were secured to the dynamometer chair using a five-point harness and seatbelt. A computer monitor provided torque feedback to the participant. To ensure no contribution of force transmitting from the upper body, participants crossed their arms over their chest during the contractions. Participants were then instructed to perform three bilaterally maximum voluntary contractions (MVCs), unilaterally three MVCs with their left leg, and three unilaterally MVCs with their right leg, where the order of testing was randomized. Participants were asked to hold the contraction for 5 s to provide sufficient time to reach maximal torque production. A two-minute rest period was given after each MVC to minimize fatigue. During all trials, experimenters provided verbal encouragement (such as “push as hard as you can”) to elicit motivation for maximal torque production.

### Surface electromyography

Skinfold measurements were taken on the right leg of all participants in a supine position for anterior thigh (mid-point between the patella and the inguinal fold) and patella (2 cm above the proximal edge of the patella). Thigh girth for the right leg of each participant was also measured. Criteria for skinfold measurements was similar to that of Kuiken et al. [15] to ensure that all participants had less than 0.4 mm of adipose tissue which otherwise would interfere with the myoelectric signal. Bipolar surface electrodes (Duotrode silver-silver chloride electrodes (Myo-tronics Inc.); interelectrode spacing of 21.0 ± 1.0 mm) were placed bilaterally (left and right) on palpated muscle bellies of the rectus femoris (RF), vastus medialis (VM), and vastus lateralis (VL) adhering to Seniam guidelines (The Seniam Project, 1999). To reduce impedance caused by skin, the area was shaved and cleaned with alcohol wipes prior to electrode placement. For the RF, electrodes were placed parallel to the muscle fibers at half the distance between the anterior superior iliac spine (ASIS) and the superior part of the patella. Electrodes were placed over the VL at two-thirds the distance between the ASIS and the lateral aspect of the patella. Electrodes were then placed over the VM at an oblique angle (55°) at 80% of the distance between the ASIS and the joint space in front of the anterior border of the medial ligament. The reference electrode was placed over the right patella. All data was filtered using commercial software (OTBioLab Software, Bioelettronica, Italy). Due to technical difficulties with the instrumentation, nine of the fourteen participants (3 men and 6 women) produced viable EMG data. Table 1 provides the detailed characteristics of all participants.

### Electroencephalography

A dry EEG headset (Cognionics Quick-30) was used to acquire continuous brain wave activity during each set of 3 trials for the leg press. The sampling rate of the EEG was 1000 Hz and conductive gel was applied when needed to keep impedances around 100 kOhm for the electrodes. The system was positioned on each participant’s head based on the standard 10/20 channel system with the left earlobe as the reference point as shown in Fig. 1.

### Data analysis

#### Torque

The trial with the highest peak torque was chosen for further analysis. The corresponding trial was used for further processing for the EMG data. The bilateral limb ratio (BLR) was calculated similar to previous studies as follows [27].

1 $$BLR_{torque} \left( \% \right) = \frac{Bilateral\,Peak\,Torque}{{Total\,Unilateral\,Peak\,Torque}} \times 100$$

#### Surface electromyography

Surface EMG signals that corresponded to the trial with the maximum peak torque were used for processing. For these trials, a bandpass filter of 20–400 Hz was applied using the OTBioLab software and then exported into an Excel spreadsheet. For further processing, the data was converted to a MATLAB file and with a notch 60 Hz filter applied. The amplitude of the EMG signal was estimated using the root mean square (RMS) calculation. A 1.0 s window of EMG data, centered at the peak torque was used for all calculations similar to previous studies [16, 17].

#### Electroencephalography

The EEG data were processed using a custom MATLAB script (MathWorks, Natick, MA, USA) using EEGLAB [4] functions. Data were first filtered with a band-pass filter of 0.1–100 Hz to eliminate low frequency noise/DC offset. All blinking and other ocular artifacts were removed from the data using an independent component analysis approach [4]. Epochs time-locked to the onset of movement were extracted from the data from − 1500 to 200 ms in order to analyze the MRCP. Similar to the EMG data, a notch 60 Hz filter was applied. The electrodes used to analyze the MRCP were over the left and right precentral cortex (C3 and C4, respectively), as they reside over the primary motor cortex [24, 25]. The grand average of all EEG trials was calculated and then used to obtain the MRCP according to [34] at 3 phases: readiness potential (RP, − 1000 to − 600 ms), negative slope (NS’; − 600 to − 200 ms) and the motor potential (MP; − 200 to − 50 ms).

#### Statistical analysis

All statistical analyses were completed using R Studio 1.0. 136 (RStudio, Boston, MA). The alpha level was set to 0.05. Normality of data was tested using a Shapiro-Wilks test prior to any statistical analyses. The torque based BLR was compared using a Student’s t-test to a ‘no deficit’ and ‘no facilitation’ condition (i.e. BLR = 100%). The BLR for a subset group with BLR < 100% was also compared to determine if the lower BLR was, in fact, a deficit compared to 100%. As the EMG data was not normal, a log transformation was performed on the positively-skewed raw EMG values in order to perform parametric statistics. A 3 × 2 × 2 mixed factorial two-way repeated measures analysis of variance (ANOVA) with Greenhouse Geisser corrections for sphericity was used to examine differences of activation of the three knee extensor muscles (RF, VL, VM) in the two legs (left, right) across the different leg press conditions (unilateral, bilateral). Post-hoc pairwise comparisons were performed using t-tests with Bonferroni corrections. For the EEG data and each component of the MRCP, paired t-tests were used to measure the within electrode differences across the unilateral and bilateral condition. Independent t-tests were used to measure differences between the left and right electrodes within each condition.

## Results

### Unilateral and bilateral torque

The mean torque data for the unilateral and bilateral conditions are shown in Table 2. The mean BLR across all participants was 94.8 ± 22.0% was not found to be statistically significantly lower than 100%. However, of the fourteen participants, 10 exhibited a bilateral limb deficit (less than 100%). An analysis was performed on both the participants that demonstrated a BLD response (n = 10) and the participants that demonstrated a facilitation (n = 4). A t-test showed that those participants that exhibited a deficit had a mean BLR = 81.4% which was significantly lower than 100% (*p* < 0.01). The participants that demonstrated a facilitation had a BLR of 117.1% which was significantly higher than 100% (*p* = 0.0155) indicating a bilateral facilitation.

**Table 2:** Torque data

	Unilateral left	Unilateral right	Bilateral	BLR (%)
Torque (Nm) (n = 16)	127.6 ± 45.2	129.1 ± 45.2	232.0 ± 61.8	94.8 ± 22.0

### Unilateral and bilateral EMG

**Fig. 2:**
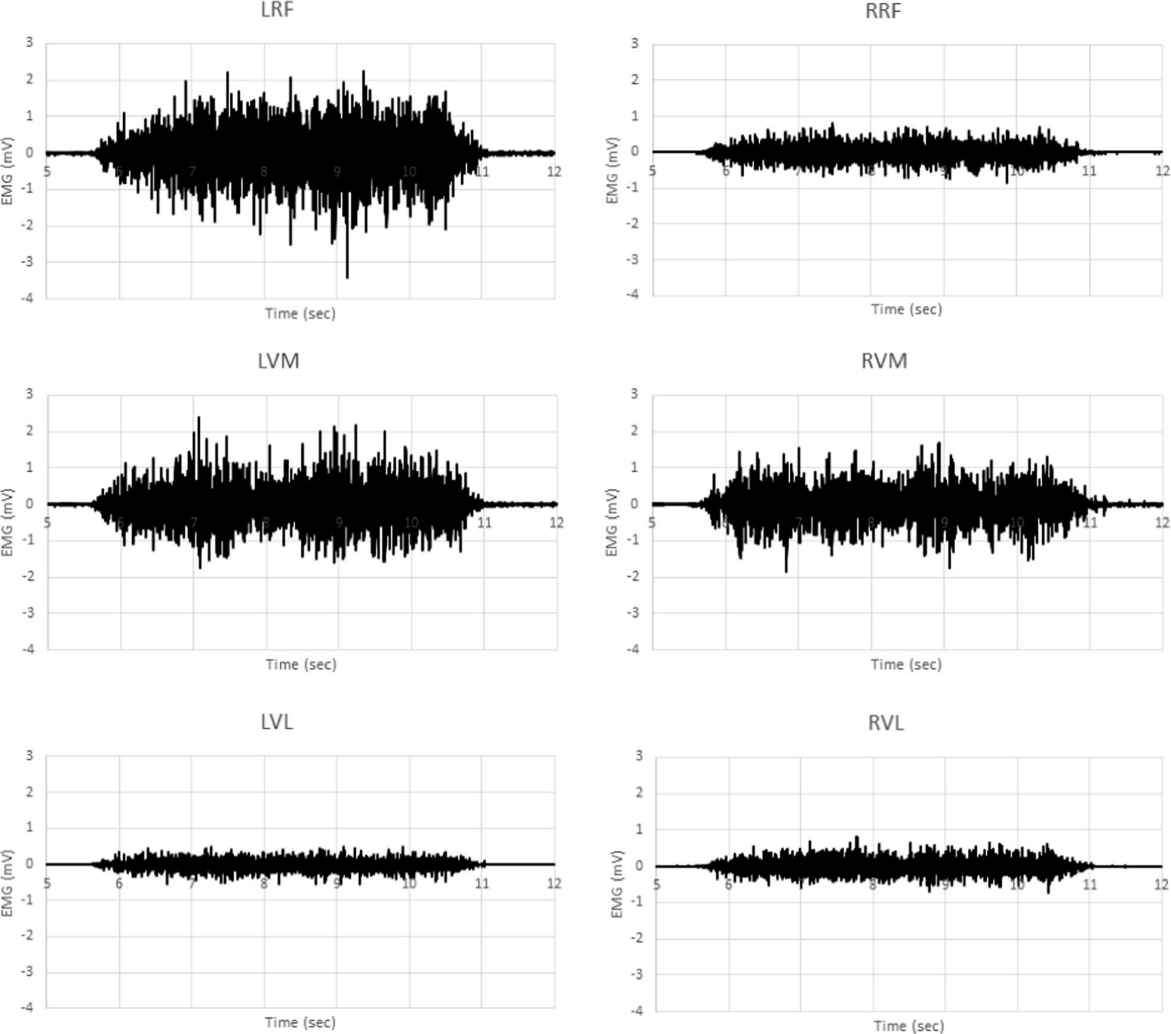
Sample Data. EMG data from one subject during an MVC in a bilateral leg press. *First column* EMG data from the left limb (RF, VM, VL). *Second column* EMG data from the right limb (RF, VM, VL)

**Fig. 3:**
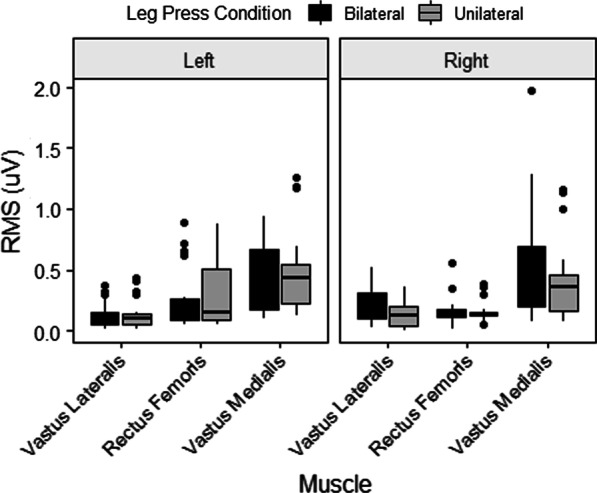
Original mean RMS values of the left and right sided muscles between the unilateral and bilateral conditions

**Fig. 4:**
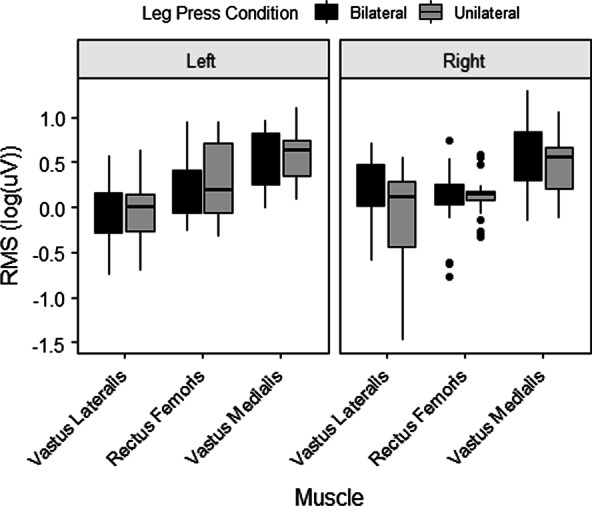
Transformed mean RMS value of the left and right sided muscles between the unilateral and bilateral conditions

Due to technical issues with the EMG system, there was incomplete data for 5 participants and therefore EMG results are presented for the remaining 9 participants. Figure 2 provides sample EMG data from one subject. Muscle activity from the rectus femoris (RF), vastus medialis (VM), and vastus lateralis (VL) during unilateral and bilateral isometric leg press is shown. Table 3 presents the amplitude data for each muscle (RF, VM, VL) for bilateral and unilateral conditions for 9 participants. The two-way repeated measures ANOVA did not reveal any significant differences due to condition (bilateral versus unilateral) (F(8) = 0.24, *p* = 0.87). The subset of individuals who presented a BLD were also examined and there were no significant differences detected due to condition in those individuals either. There were significant differences detected between muscles (F(8) = 10.14, *p* < 0.001) with the right VM having significantly higher amplitude for the unilateral right (0.347 ± 0.318 mV) and bilateral right (0.436 ± 0.470) conditions, respectively) than either the VL or RF (F(16) = 0.23, *p* < 0.05). The VL had significantly lower amplitudes in all conditions (0.127 ± 0.138; 0.111 ± 0.104; 0.120 ± 0.105; 0.162 ± 0.147 for unilateral left, bilateral left, unilateral right, and bilateral right, respectively). Figure 3 provides the original mean RMS data of each muscle under each condition for both the left and right side while Fig. 4 presented the log-transformed data.

**Table 3:** EMG data

Condition	Muscle EMG Activity (mV), n = 9		
	RF	VL	VM
Unilateral left	0.305 (0.294)	0.127 (0.138)*	0.358 (0.302)
Bilateral left	0.278 (0.286)	0.111 (0.104)*	0.360 (0.280)
Unilateral right	0.179 (0.134)	0.120 (0.105)*	0.347 (0.318)†
Bilateral right	0.184 (0.139)	0.162 (0.147)*	0.436 (0.470)†

Values are in mean (SD)

*Indicates values that are significantly lower

†Indicates values that are significantly higher

### Unilateral and bilateral EEG

**Fig. 5:**
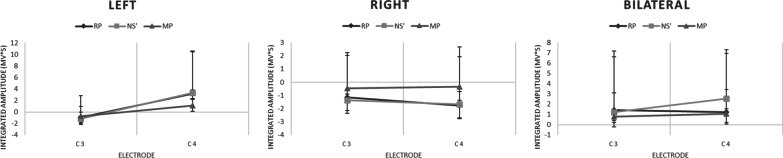
Mean integrated amplitudes (mV*s) of the RP, NS’, and MP at the C3 and C4 electrodes during each condition

Figure 4 illustrates the average integrated amplitudes during the three components of the MRCP (RP, NS’, and MP) during the three conditions. The values displayed represent the magnitude of the MRCP components at the left (C3) and right (C4) precentral cortex, respectively. When looking for asymmetries in magnitude between the two hemispheres, there were no significant differences found between the left and right side for the RP, NS’, or MP for any condition (*p* > 0.05). When comparing within each electrode, the average NS’ amplitude recorded from the precentral right hemisphere (C4) was significantly greater (t(7) = 3.37, *p* < 0.05) in the bilateral condition compared to the unilateral right condition (0.00257 ± 0.00475 mV*s and −0.00168 ± 0.00361 mV*s for the bilateral and unilateral right conditions, respectively). The average MP in the C4 electrode was also greater (t(7) = 2.68, *p* < 0.05) in the bilateral condition compared to the unilateral right condition (0.00106 ± 0.00238 mV*s and −0.000351 ± 0.00187 mV*s for the bilateral and unilateral right conditions, respectively) (Fig. 5).

## Discussion

This pilot study presents BLD leg press similar to other studies [11, 21, 22], but with varying results. The mean BLR detected in the present study was 94.8 ± 22.0%, with the mean BLR of individuals that incurred a deficit being ~ 81%. Of the participants that did present with the deficit, this value is slightly higher than what was discovered in previous research [21], however the sample in the present study was much smaller. MacDonald et al. studied varsity swimmers and their results indicated a lower BLD than our results, possibly due to the type of training completed by the athletes, i.e. both unilateral and bilateral exercises. In contrast, a post-study survey revealed that, in our study, only six of the 10 participants that presented a deficit performed traditional bilateral leg press training on a regular basis (1–2 times a week), which would explain, at least partially, our relatively lower BLD [18]. Furthermore, the average torque data of the sample presented in this work was also higher than that of MacDonald et al. [21] which may have been affected by bilateral leg training. The differences of the training practices between our sample population and that of MacDonald et al. [21] may have reduced the overall BLD effect seen in our results.

It has been previously reported that the BLD is more evident in dynamic exercises (e.g. isokinetic knee extension) than isometric contractions [10, 16, 18]. Similar to Janzen et al. [11], we found that the BLD is present in complex exercises such as the leg press which combines hip and knee extension. In addition, the nervous system may be more involved during multi-articulate contractions such as the leg press, that involve movements at multiple joints (Chilibeck et al., 1998). Magnus and Farthing [22] suggested that exercises involving multiple muscle groups, such as the leg press, might exhibit larger bilateral deficits because it is more difficult to maintain postural stability under the bilateral condition. MacDonald et al. [21] further examined the postural stability theory and found that while a BLD was present during bilateral isometric leg press there was no deficit during bilateral handgrip exercises for athletic and non-athletic women. In the present study handgrip was not recorded. There is comparable evidence to suggest that single-jointed movements, such as knee extension, may result in a smaller bilateral deficit compared to multi-jointed movements, such as a lateralis pull-down and leg press [11]. This is because multi-jointed movements tend to involve larger muscles and greater force production, thus requiring greater postural stability. It was determined that muscle activation of the trunk was significantly greater in the leg press, a multi-joint movement, compared to the knee extension and handgrip exercises, which are single-jointed movements [35].

The surface EMG data in this study did not show any differences between the bilateral and unilateral conditions for any of the quadriceps muscle. The EMG was also examined from those that exhibited a BLD (10/14 participants) and no significant trend was observed with respect to the deficit. Similar to previous studies, this study found that the muscles of the quadriceps femoris are not homogeneously activated during the leg press [5]. They studied knee extension and leg press at differing intensities and found inter-muscle and inter-exercise differences in the activation of the quadriceps femoris from the involvement of the hip extension torque and that the RF activation is low in multi-joint exercise. However, Alkner et al. [1] did not find significant differences in the EMG amplitude of the VL, VM, RF and Biceps Femoris (BF) between isometric knee extension and leg press. While there were no statistically significant differences detected between the bilateral and unilateral conditions, there is potential for discrepancies in homogenous muscle activation during leg press; therefore, future work should include larger sample sizes to illustrate the possible effect. In addition, due to the loss of some participants’ EMG data, the majority of the remaining EMG data were from female participants which may have also influenced the results. One limitation of the present study was the lack of measured antagonist muscle activity. In addition, this work used traditional bipolar surface EMG over the three muscles. Using multichannel, high density EMG electrodes over the entire quadriceps muscle may reveal greater insight regarding muscle activation during the leg press and also provide greater support to the postural stability theory of the BLD.

Previous studies that have investigated the role of surface EMG and the development of the BLD have been inconclusive and in many cases EMG data have not paralleled force or torque data under the same conditions. Some researchers have reported that the amplitude of the EMG signal is lower under bilateral conditions compared to unilateral conditions [3, 12, 14, 16, 21, 23, 26, 27, 31, 38, 41, 42]. Several authors [6, 23, 26] have observed a greater force reduction in the dominant limb when investigating BLD, however, these results were primarily based on upper limbs. Other studies have also found that bilateral EMG amplitudes are lower than the unilateral [3, 24, 25, 30, 43]. While some researchers have found that EMG amplitudes are lower during bilateral conditions compared to unilateral conditions [3, 12, 14, 16, 21, 23, 26, 27, 38, 42, 43] others have shown no deficit in the EMG data [8, 28, 33]. Only one movement was examined in the present study (maximal leg press) and EMG measurements were taken from the quadriceps muscle. There may have been contribution from both the hamstrings and gluteus muscles which may have provided greater insight regarding muscle activation. In addition, this study only found differences in bilateral and unilateral EMG on the left side suggesting other factors contribute to the deficit. It has been suggested by researchers that the deficit may be caused by significant decreases in motor unit activation of the quadriceps muscles during the bilateral contraction compared to the unilateral [42], decreased cortical activity [24], and a reduction in neural drive, in conjunction with interhemispheric inhibition [3, 30].

While some studies have proposed that BLD is due to neural inhibition during bilateral compared to unilateral tasks [42], few studies have used EEG to explore brain activity during these types of contractions [24]. While limited in sample size, in this pilot study we examined strength, surface EMG measures, and brain activity during bilateral and unilateral contractions. Previously, Oda and Moritani [24] concluded that there was a greater MRCP deficit of the non-dominant right hemisphere compared to the dominant left hemisphere. It was also suggested that the bilateral deficits in the integrated amplitudes for both the negative slope (NS’) and motor potential (MP) could be due to the decreased neural activation of the primary motor cortex. Similar to their findings, our results illustrated no differences between hemispheres during each condition (unilateral versus bilateral). We did note a decrease in brain activity in the (non-dominant) right hemisphere only during the unilateral right condition compared to the bilateral condition. Given that the right hemisphere controls the left side of the body, it is plausible that this hemisphere would display a decrease in neural activity when the left leg is not involved in the MVC.

This study was limited to one movement and it would be interesting to determine if there are neural differences in other types of contractions which have demonstrated the BLD such as elbow flexion. Given that the lower-extremity primary motor cortex is located in close proximity to the medial longitudinal fissure may introduce barriers in measuring interhemispheric interactions in lower extremities [29]. One such challenge may be because the electrical fields created by the activation in the adjacent parts of the fissure may be polar opposites, thus canceling out the signal when measuring the overall potential using EEG. Lastly, the loss of EMG data for five participants and the overall lower number of participants also limited the interpretability and extrapolation of the results.

## Conclusions

This pilot study found the presence of the BLD during isometric leg press. There was no evidence of reduced muscle activity in bilateral compared to unilateral contractions. There were also no significant differences found between cortical hemispheres between bilateral and unilateral contractions, indicating that the deficit was not induced because of interhemispheric inhibition during isometric leg press. This study examined contractions from healthy, university aged men and women. In this study we were able to successfully measure strength, EMG and EEG simultaneously, however the results should be interpreted with caution because of the limited sample. A higher sample size as well as a larger age range may provide greater information regarding muscle and neural adaptation due to the deficit. Furthermore, it has been shown that the BLR can be reduced with targeted training. Including EEG measurement may provide greater insight regarding the response of the deficit to training.

## Data Availability

The datasets generated and/or analysed during the current study are not publicly available due privacy issues but are available from the corresponding author on reasonable request.

## Abbreviations

ANOVA: Analysis of variance
ASIS: Anterior superior iliac spine
BLD: Bilateral limb deficit
BLR: Bilateral limb ratio
BP: Bereitschaftspotential
EEG: Electroencephalography
EMG: Electromyography
MRCP: Movement related cortical potential
MP: Motor potential
MVC: Maximal voluntary contraction
NS: Negative slope
RF: Rectus femoris
RMS: Root mean square
VL: Vastus lateralis
VM: Vastus medialis

## Code availability

The software and custom code used are not available for public use.
